# Head-to-head comparison of ^68^Ga-PSMA-11 and ^18^F-FDG in delayed PET/CT imaging in prostate cancer diagnosis

**DOI:** 10.3389/fonc.2025.1515653

**Published:** 2025-04-11

**Authors:** Biyao Hu, Haonan Yu, Meijie Pan, Hailei Yang, Xiling Xing, Dong Li, Shaobo Yao, Qiusong Chen

**Affiliations:** ^1^ Department of PET/CT Diagnostic, Tianjin Key Lab of Functional Imaging & Tianjin Institute of Radiology, Tianjin Medical University General Hospital, Tianjin, China; ^2^ The Clinical Research and Translational Center, The First Affiliated Hospital of Fujian Medical University, Fuzhou, Fujian, China; ^3^ Department of Radiology, Tianjin Medical University General Hospital, Tianjin, China

**Keywords:** prostate cancer, PET/CT, ^68^Ga-PSMA-11, ^18^F-FDG, delayed imaging

## Abstract

**Purpose:**

Delayed PET/CT imaging with ^68^Ga-PSMA-11 is valuable in the detection of primary prostate (PCa) lesions and the differentiation of suspicious lesions. However, ^18^F-FDG PET/CT has been overlooked due to its low sensitivity to PCa during routine examination. This study aimed to compare the clinical impact of PSMA and FDG in delayed PET/CT imaging in PCa diagnosis.

**Methods:**

Between 2019 and 2024, 65 PCa patients who underwent early (1 h post-injection) and delayed (3 h p.i.) PSMA and FDG scans were retrospectively analyzed. The delayed scans were conducted to clarify unclear findings in early scans or to increase the tumor lesions uptake in negative early scans. All patients were asked to drink 1 L of water between early and delayed scans. The number of primary and metastatic lesions, sensitivity, specificity, diagnostic accuracy, lesions changes in SUV_max_ of early and delayed scans were evaluated. Correlation between SUV_max_ and Gleason score as well as SUV_max_ and PSA for PCa primary lesions diagnosis were analyzed.

**Results:**

Overall, 83 and 84 lesions characteristic for PCa in 65 patients clearly presented at 1 h and 3 h p.i. in PSMA scans, respectively. 30 and 45 lesions characteristic for PCa in 65 patients clearly presented at 1 h and 3 h p.i. in FDG scans. The 3-hour delayed imaging of FDG found more primary foci than 1-hour imaging but was much less able to detect metastatic foci than PSMA. PSMA was more sensitive than FDG in delayed imaging (96.15% vs. 84.21%), and the diagnostic accuracy for primary foci was higher for PSMA than FDG in delayed imaging (83.87% vs. 73.91%). However, FDG delayed imaging greatly improved the diagnostic accuracy for primary PCa compared to early imaging (73.91% vs.53.33%). PSMA SUV_max_ of both 1 h and 3 h p.i. were correlated with the Gleason score PSA, but FDG SUV_max_ only showed a correlation with PSA at 3 h p.i.

**Conclusion:**

PSMA PET/CT at 3 h p.i. detected the most lesions characteristic of primary PCa, and it showed higher uptake and contrast than FDG. However, to some extent, FDG delayed PET/CT imaging is still important in primary PCa diagnosis, particularly in hospitals without PSMA.

## 1 Introduction

Prostate cancer (PCa) is one of the most common malignant tumors in men, and early detection and accurate diagnosis have great significance in improving the treatment effect and survival rate of patients (1, 2).However, conventional imaging techniques, including MRI and CT (3), have limited sensitivity. In recent years, ^68^Ga-PSMA-11 PET/CT, an emerging non-invasive imaging method, has shown potential application in the diagnosis, staging, and restaging of PCa (4–7). Many studies have reported that the detection efficiency of PSMA PET is higher than that of conventional imaging methods (4, 8). In particular, PSMA delayed imaging 3 h postinjection (p.i.) has proven to be a valuable method to clarify unclear findings on routine scans at 1 h p.i. or to find new PCa lesions (9).

^68^Ga-PSMA-11 demonstrates clear advantages in the diagnosis of prostate cancer, especially in the precise detection and staging of metastatic and localized disease. ^68^Ga-PSMA-11 excels in detecting lymph node and distant metastases (e.g., bone, visceral metastases), providing more accurate staging information (10, 11). For localized prostate cancer, ^68^Ga-PSMA-11 helps determine the precise location and extent of the tumor, guiding surgical or radiotherapy planning (12). While FDG has limited utility in prostate cancer.

However, PSMA PET is owned only in some large hospitals, much more hospitals only have ^18^F-FDG for routine clinical diagnosis (13). Most studies reported that FDG has low sensitivity in detecting primary PCa (14). However, recent studies showed that FDG PET/CT were positive in patients with negative PSMA PET/CT findings (15, 16). In addition, a study confirmed increased sensitivity in FDG delayed (3h p. i.) PET/CT imaging of PCa (17). Therefore, a comparison of FDG and PSMA in delayed PET/CT imaging in PCa were needed.

At our institute, additional delayed scans have been subsequently used to increase the detection rate of PCa with FDG and PSMA in men with unclear findings 1 h p.i. In this study, we had performed a direct head-to-head comparison of FDG and PSMA delayed PET/CT imaging in PCa diagnosis.

## 2 Materials and methods

### 2.1 Patient selection

From October 2019 to April 2024, 118 patients received both ^18^F-FDG PET/CT and ^68^Ga-PSMA-11 PET/CT at Tianjin Medical University General Hospital. 53 of these patients who had been diagnosed with PCa and were treated and then revisited were excluded, and 65 patients with suspected PCa at first diagnosis were finally enrolled in our study. The following were the inclusion criteria: (a) patient suspected of possible PCa on other imaging tests such as MR or ultrasound; (b) patients had PSA laboratory results within the last two weeks; (c) FDG and PSMA-11 PET/CT examinations were performed at intervals of < 48 hours; and (d) there was no previous treatment related to PCa. The Ethics Committee of Tianjin Medical University General Hospital approved this retrospective study, and each patient signed an informed consent form. The patients’ characteristics are presented in **Table 1**. 65 patients in this study received both 1 h and 3 h p.i. delayed imaging with PSMA. 64 received 1 h imaging with FDG and 55 received 3 h p.i. delayed imaging with FDG. 29 of these patients were followed up with pathology of the prostate.

**Table 1:** Patients and tumor characteristics.

Characteristics	Date
No. of patients	65
Median age	70.5 (IQR, 66-75)
Median PSA (ng/mL)	12.3 (IQR, 7.3-20.6)
Initial PSA (ng/mL)	
≤ 10	22 (34%)
10 – 20	25 (38%)
≥520	18 (28%)
Gleason score	
< 6	5 (17%)
6	6 (21%)
7	12 (41%)
8	4 (14%)
9	2 (7%)
Risk factors	
smoking	32 (49.2%)
alcohol use	29 (44.6%)
diabetes	25 (38.5%)
family history of cancer	NA
obesity	NA
Demographic	NA

IQR, interquartile range; PSA, prostate-specific antigen level.

### 2.2 ^18^F-FDG and ^68^Ga-PSMA-11 PET/CT

Patients were fasted for 6 h before receiving FDG injection, at a dose of 5.55 MBq/kg, and blood glucose should be controlled below 11.1 mmol/L. PET/CT (GE Discovery 710, GE Healthcare, USA) scanning was performed 1 h p.i., and CT images (3.75 mm slice thickness, automatic mA current:120kV, Scan Type: Helical, Rotation time:0.8, Rotation Length: Full, Pitch & Speed: 1.375:1&55.00) were scanned from the upper thigh to the skull. PET scanning time for each bed was 2 min in 3-dimensional mode, and the images were reconstructed in a 192×192 matrix with a pixel size of 3.27 mm. The reconstruction method was VUE Point FX (GE Healthcare), which uses time-of-flight information and includes a fully 3-dimensional ordered-subsets expectation maximization algorithm with 2 iterations, 24 subsets, and a filter cutoff of 6.4 mm. The quantitation method was SharpIR. A standard Z-filter was applied to smooth between transaxial slices. The Patients undergoing ^68^Ga-PSMA PET/CT were not required to fast. PSMA was injected at a dosage of 1.85 MBq/kg, and PET/CT was performed 1 h p.i. CT images (CT parameters are consistent with those used for FDG) were scanned from the upper thigh to the skull. PET scanning time at each bed was 3 min. The same reconstruction parameters were used for PSMA. All patients were instructed to drink 1 L of pure water after the first examination and were encouraged to urinate several times. Delayed scanning was performed at 3 h p.i., and the scanning range of delayed imaging for FDG PET/CT was the pelvis, with a scanning time of 3 min per bed. The range of delayed imaging for PSMA PET/CT was generally the pelvis. If suspected metastatic foci were detected during the first imaging, delayed scanning could be performed with a scanning time of 5 min per bed. In order to better visualize the lesion, we used higher CT parameters in delayed imaging, the details of which are as follows:1.25mm slice thichness;300mA current;120Kv; The Reconstruction Algorithm was Q.AC. All patients were examined on the same scanner.

### 2.3 Image evaluation

Two nuclear physicians with more than 10 y of PET/CT experience, on a computer-assisted reading application (AW Server, version 4.6; GE Healthcare), read all datasets independently and resolved any disagreements by consensus. Lesions suggestive of PCa observed with the naked eye were counted and analyzed for localization (primary foci, lymph nodes [LN], bone and soft tissue metastases) according to interpretive guidelines (18–21). Volumes of interest (VOI) were plotted around positive lesions and the maximum standardized uptake value (SUV_max_) of each spherical VOI was measured. For calculation of the SUV_max_, positive PSMA and FDG were defined as focal avidity greater than the background in the mediastinal blood pool after exclusion of other important pitfalls or physiological uptake of the two radiotracers. Circular regions of interest were drawn around areas with focally increased uptake in transaxial slices and automatically adapted to a 3-dimensional volume of interest at a 70% isocontour as previously described. For patients with negative test results, we measured the SUVmax of the entire prostate to be counted in the statistics. PSA measurements, imaging examination (including CT, MRI, whole-body bone scan, or PET/CT re-examination), and biopsies were used for follow-up. We used composite validation including histopathology, PSA decreases after PET-directed radiotherapy, and follow-up imaging to verify these positive results.

### 2.4 Statistical analysis

Results are shown as mean ± standard deviation or frequency (%). The Wilcoxon signed-rank tests were used to compare values of baseline and delayed imaging (SUV_max_ of FDG and PSMA lesions). Statistical significance of the association between positive/negative findings on FDG PET/CT and Gleason score was assessed by using the chi-squared test, where applicable. For PSMA we used the same method of data analysis. Unpaired two-tailed t-test was used to compare patients with FDG and PSMA SUV_max_ in terms of PSA level or Gleason score. PET uptake and PSA level or Gleason score were compared by use of the Spearman correlation. A P value of < 0.05 was considered statistically significant. All statistical analysis was performed using SPSS software (version 26.0; SPSS Inc.) or GraphPad Prism (version 9.0.0; GraphPad Software).

## 3 Result

### 3.1 Characteristics of enrolled patients with PCa

A total of 118 patients were retrospectively analyzed between October 2019 and April 2024, of which 53 patients were diagnosed after surgery as well as after radiotherapy and were excluded from the study. 65 patients, diagnosed by puncture or suspected to have probable PCa by other imaging methods and were not treated in any way, were recruited in this retrospective analysis. The clinical characteristics of patients were shown in **Table 1**. The median PSA was 12.3 ng/mL (interquartile range [IQR] 7.3-20.6), 65 (100%) of the patients underwent PSMA 1 h p.i. conventional imaging and 3 h delayed imaging. 64 (98%) patients underwent FDG 1 h p.i. conventional imaging and 55 (85%) underwent 3 h p.i. delayed imaging. 65 (100%) patients had PSA results, and 29 (45%) patients had pathologic puncture results, of which 24 (83%) had Gleason scores ≥ 6 and 5 (17%) were negative.

### 3.2 Detection of lesions by FDG and PSMA PET/CT

**Figure 1:**
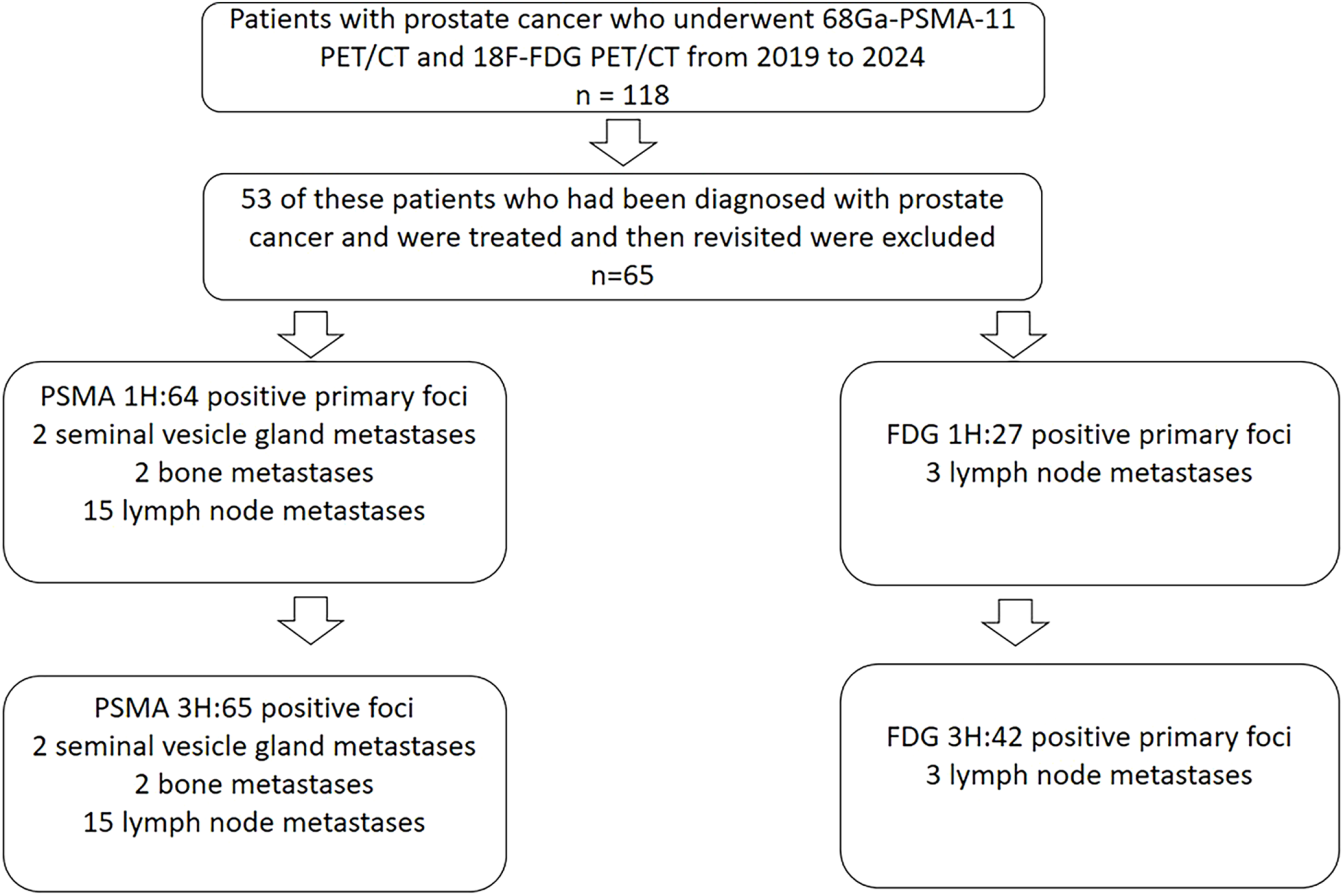
Patient recruitment flowchart.

A total of 64 PSMA positive primary foci were found on 1 h p.i. conventional imaging and 65 positive foci were found on 3 h p.i. delayed imaging. There were 2 cases of seminal vesicle gland metastases, 2 bone metastases, and 15 lymph node metastases. All metastases were visible on conventional imaging at 1 h p.i. imaging and 3 h p.i. delayed imaging in PSMA. A total of 27 positive primary foci were found in 1 h p.i. routine FDG, 42 positive primary foci were found in 3 h p.i. delayed FDG, 3 lymph node metastases were found in 1 h and 3 h p.i. (**Figure 1**), and 31 foci had pathologic results, of which 26 were diagnosed with prostate cancer, and 5 were negative. 2 patients with PSMA-positive foci did not correspond to the location of FDG-positive foci. 1 patient with PSMA-negative results had positive pathology and FDG delayed results. 2 patients had positive PSMA results but the pathology and FDG results were negative.

### 3.3 The sensitivity, specificity, diagnostic accuracy of FDG and PSMA for prostate cancer primary foci

We compared the sensitivity specificity and accuracy of 1 h conventional and 3 h delayed imaging of FDG and PSMA in the diagnosis of primary foci of PCa. The sensitivity of PSMA is higher than that of FDG both in the 1 h conventional and 3 h delayed imaging, but there is no advantage of PSMA in specificity (**Table 2**). In terms of accuracy, 1 h image of PSMA was slightly higher than the 3 h delayed imaging (87.10% vs. 83.87%). However, delayed imaging of FDG had a significant improvement in diagnostic accuracy as compared to conventional imaging (73.91% vs. 53.55%).

**Table 2:** Sensitivity-specificity and accuracy of FDG vs. PSMA in the PCa diagnosis.

	Sensitivity	Specificity	Accuracy
FDG (1-hour)	52% (13/25)	60% (3/5)	53.33% (16/30)
FDG (3-hour)	84.21% (16/19)	25% (1/4)	73.91% (17/23)
PSMA (1-hour)	100% (26/26)	20% (1/5)	87.10% (27/31)
PSMA (3-hour)	96.15% (25/26)	20% (1/5)	83.87% (26/31)

### 3.4 Changes in SUV_max_ at two time point of FDG and PSMA

We measured SUV_max_ values of positive prostate foci in all patients (if the patient was negative, we measured SUV_max_ values of the whole prostate) both at 1 h and 3 h. On comparative analysis, the mean of PSMA SUV_max_ at 3 h p.i. was 18.16 (95% CI 14.73 – 21.60), which was significantly higher than the mean of SUV_max_ at 1 h p.i. 13.3 (95% CI 10.55 – 16.05), *P* < 0.0001. The same result was seen in FDG imaging, the mean of SUV_max_ at 3 hours was 6.13 (95% CI 5.00 – 7.25), which was significantly higher than the mean of SUVmax at 1 h p.i. 4.49 (95% CI 3.79 – 5.19), *P* < 0.0001. Delayed imaging improved the visibility of the lesions both in PSMA and FDG imaging (**Table 3**).

**Table 3:** Changes in lesion SUV_max_ at 1 and 3 h.

Imaging modality	No. of lesions	1-hour (normal imaging)		3-hour (delayed imaging)		*P* value
		SUV_max_	95% confidence interval	SUV_max_	95% confidence interval	
^68^Ga-PSMA	68	13.30	10.55 - 16.05	18.16	14.73 - 21.60	< 0.0001
^18^F-FDG	55	4.49	3.79 - 5.19	6.13	5.00 - 7.25	< 0.0001

**Figure 2:**
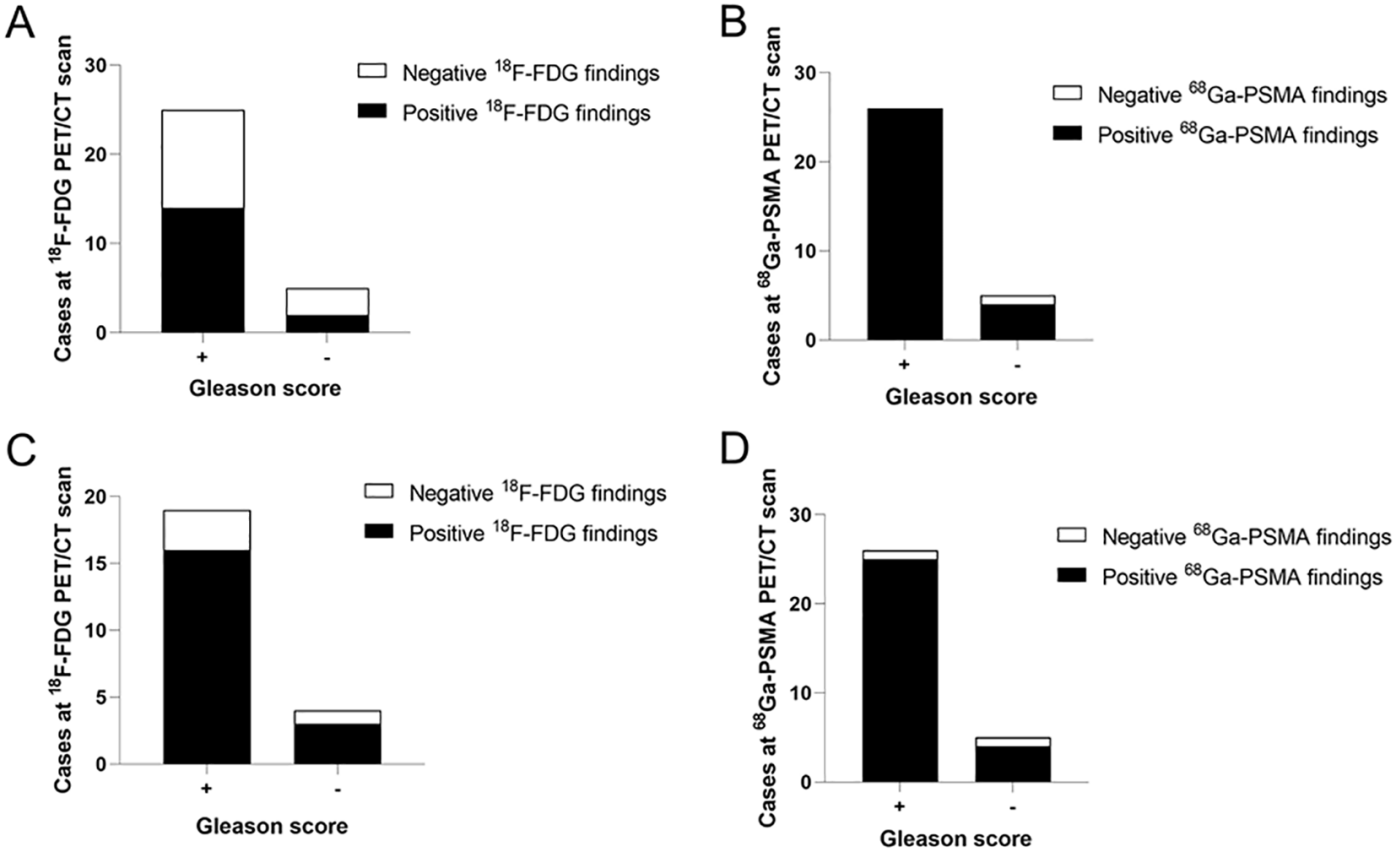
Analysis the relationship between cases at two types of imaging and pathological results. **(A)** FDG detection rate of PCa at 1 h was lower in the “Gleason score +” than in the “Gleason score -” group (56% vs. 60%). **(B)** The “Gleason score +” group had a higher PSMA detection rate than the “Gleason score -” group at 1 h p.i. (100% vs. 20%). **(C)** FDG detection rate of PCa at 3 h p.i. was higher in the “Gleason score +” than in the “Gleason score -” group (84.21% vs. 25%). **(D)** The “Gleason score +” group had a higher PSMA detection rate than the “Gleason score -” group at 3 h p.i. (96.15% vs. 20%). “+” mean Gleason score >= 6, “-” mean Gleason score < 6.

61 of the 64 positive primary foci identified in the 1 h image of PSMA had an increased SUV_max_ in the 3 h delayed image, and 3 positive foci SUV_max_ decreased. Only 27 positive foci were observed in the 1 h image of FDG, 26 positive foci had an increased SUV_max_ in the 3 h delayed image and an additional 16 positive foci with increased SUV_max_ were identified. After comparing the pathological results, we concluded that the 3 h delayed imaging significantly improves the diagnostic efficiency of FDG in the primary foci (**Figure 2**).

### 3.5 Correlation analysis of primary lesions SUV_max_ with PSA and Gleason score

**Figure 3:**
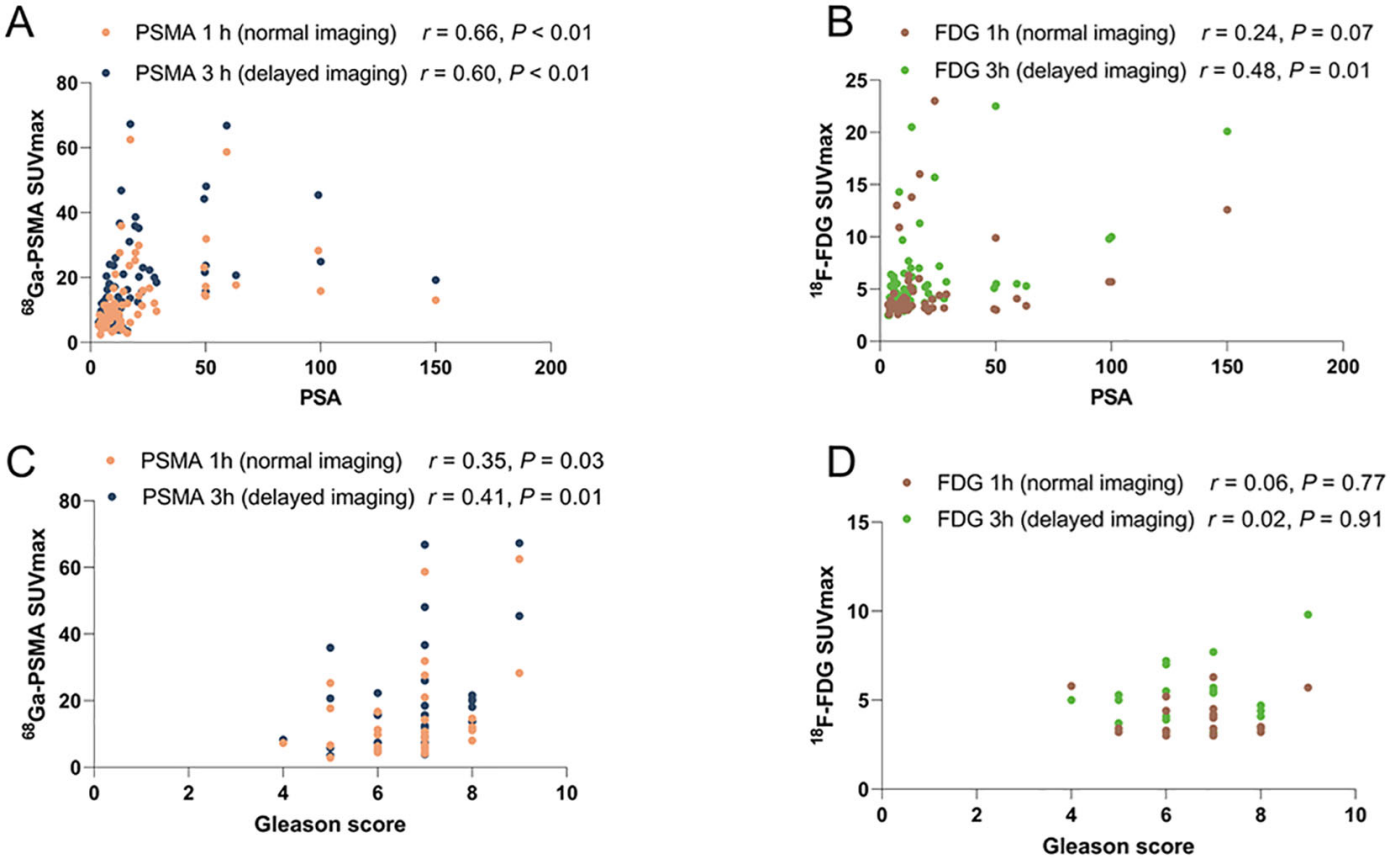
The association between PET uptake intensity and PSA or Gleason score. **(A)** Spearman r for PSMA 1 h SUV_max_ and PSMA 3 h SUV_max_ by PSA. Significant Correlation with PSMA 1 h p.i. (n = 68) (*r*, 0.66; *P* < 0.001); PSMA 3 h p.i. (*r =*, 0.60; *P* < 0.01). **(B)** Moderate correlation between SUV_max_ and PSA for FDG (n = 59; 1 h, *r* = 0.24; P = 0.07 3 h, *r* = 0.48; P=0.01). **(C)** Moderate correlation between SUV_max_ and Gleason score in PSMA 1 h p.i. (n = 68) (*r* = 0.35; *P* = 0.03); PSMA 3 h p.i. ( *r* = 0.41; P = 0.01). **(D)** No correlation between SUV_max_ and Gleason score for FDG (n = 59; 1 h, *r* = 0.06; 3 h, *r* = 0.02).

The relationship between PSMA uptake intensity and PSA expression and the comparison of PSMA uptake values with histopathological Gleason scores are shown in **Figure 3**. There was a significant correlation between SUV_max_ and PSA in samples obtained by PSMA 1 h p.i. (n = 68) (*r*, 0.66; *P* < 0.001). And there was also a significant correlation between SUV_max_ and PSA for samples (n = 68) obtained by PSMA 3 h p.i. (*r =*, 0.60; *P* < 0.01). A moderate correlation was detected between SUV_max_ and Gleason score for PSMA (1 h, *r* = 0.35; 3 h, *r* = 0.41) (**Table 4**).

**Table 4:** Correlation of primary foci SUVmax with PSA and Gleason scores.

Imaging modality	No. of lesions	1-hour (normal imaging)			3-hour (delayed imaging)		
		*r*	95% confidence interval	*P* value	*r*	95% confidence interval	*P* value
^68^Ga-PSMA							
PSA	68	0.66	0.49 - 0.78	< 0.01	0.60	0.42 - 0.74	< 0.01
Gleason score	31	0.35	-0.02 - 0.63	0.03	0.41	0.05 - 0.67	0.01
^18^F-FDG							
PSA	59	0.24	-0.02 - 0.46	0.07	0.48	0.26 - 0.66	< 0.01
Gleason score	23	0.06	-0.37 - 0.47	0.77	0.02	-0.40 - 0.44	0.91

As shown in **Figure 3**, a moderate correlation between SUV_max_ and PSA was observed in FDG 1 h p.i. (n = 59; *r* = 0.24; *P* = 0.07). A moderate correlation between SUV_max_ and PSA was observed in FDG 3 h p.i. (n = 59; *r* = 0.48; *P* < 0.01). Strong correlation between SUV_max_ and PSA was detected for the samples obtained (n = 59; *r* = 0.48; *P* < 0.01). However, no correlation was observed between SUV_max_ and Gleason score for FDG (1 h, *r* = 0.06; 3 h, *r* = 0.02) (**Table 4**).

## 4 Discussion

The limitations of FDG in the diagnosis of prostate cancer can be confirmed from our study, especially in detecting prostate cancer lesions, specifically addressing the low sensitivity and specificity. However, 3 h delayed imaging did significantly improve the accuracy of FDG for prostate cancer diagnosis. It provides an excellent complement to FDG for the diagnosis of prostate cancer. In the past, many studies have confirmed that PSMA PET/CT delayed imaging is of great help in the diagnosis of PCa (9), especially in the detection and diagnosis of metastatic foci. However, due to the fact that the sensitivity of FDG is not very high in PCa it has been neglected (14), and few studies were performed to confirm the usefulness of FDG delayed imaging. In this study, comparison of PSMA and FDG imaging proved that delayed imaging of FDG can be of great help in detection PCa primary foci.

**Figure 4:**
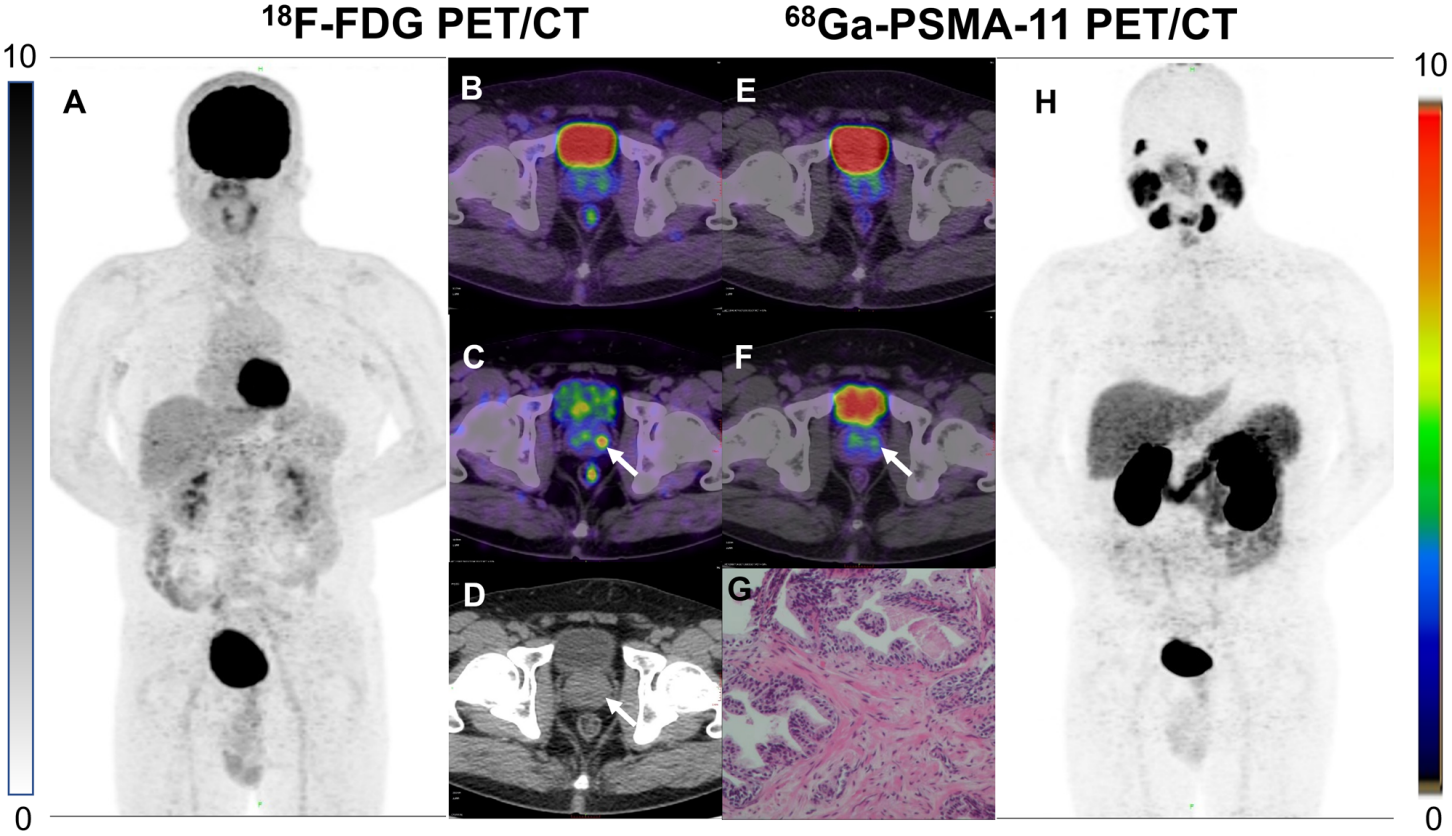
**(A)** 53-year-old man with PCa, no abnormal uptake of the prostate (SUV_max_, 3.9) was seen on FDG 1 h conventional imaging **(B)**, and a high uptake foci (SUV _max_, 6.2) was seen at FDG 3 h delayed imaging on the left side of the prostate [**(C)**, white arrows]. H&E staining results proved this early PCa **(G)**. No abnormal uptake was seen at PSMA 1 h and 3 h PSMA delayed imaging **(E, F)**. No obvious lesions were seen on the CT images either **(D)**. **(A, H)** are MIP maps of whole-body PET scans of FDG and PSMA, respectively.

**Figure 5:**
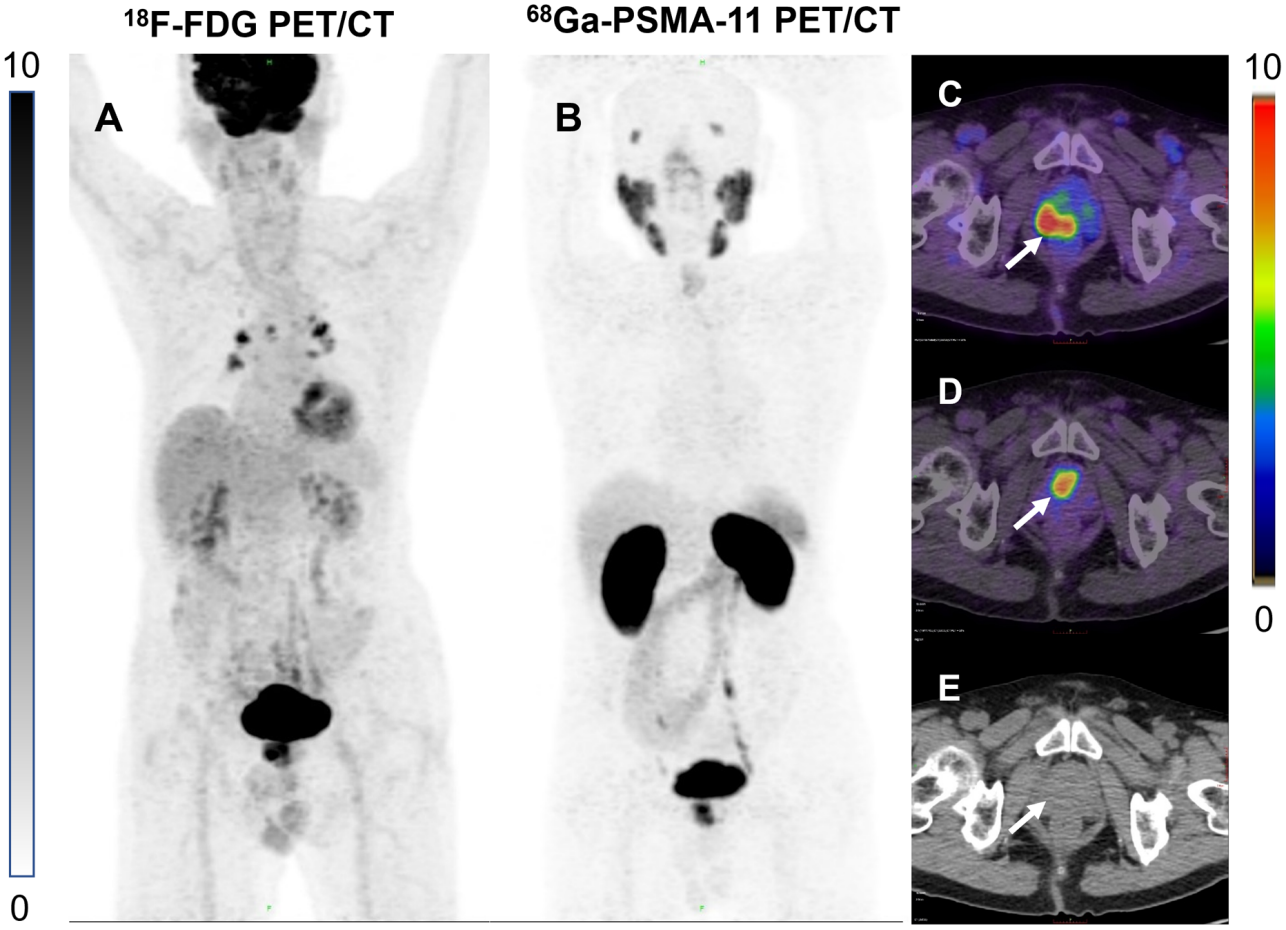
**(A)** 86-year-old man with a high uptake lesion in the right posterior aspect of the prostate was detected at FDG 3 h delayed imaging [**(C)**, white arrows]. High uptake lesion in the central anterior aspect of the prostate was detected on PSMA 3 h delayed imaging [**(D)**, white arrows]. No obvious lesions were seen on the CT images either **(E)**. **(A, B)** are MIP maps of whole-body PET scans of FDG and PSMA, respectively.

In addition, we found some interesting cases. A patient with negative PSMA results but positive pathology and FDG results (**Figure 4**), which may be explained by some recently reported studies. Paschalis et al. observed inter-patient heterogeneity in PSMA expression, a significant number of PCa patients have undetectable PSMA expression (22). One study confirmed that PCa patients with suppressed PSMA expression have high expression of glucose transporter enzyme (23). These findings have been confirmed by numerous studies and cases (24–28). Another case with positive results with FDG and PSMA showed inconsistent lesions sites, which may be related to the heterogeneity of PSMA expression within the tumor (**Figure 5**) (29, 30).

In this study delayed FDG PCa imaging was showed a diagnostic accuracy of 73.91%. Because 5 patients diagnosed with PCa had positive results in the 1 h imaging with FDG, we did not perform the 3 h delayed imaging also considering the patient’s age and physical limitations. If the 3 h delayed imaging results of these 5 patients were positive, the sensitivity and accuracy of FDG delayed imaging might be better. We concluded that 3 h delayed FDG imaging is of significant in the detection of PCa primary foci.

The associated increased uptake of nutrients, including glucose, had been clearly demonstrated in PCa in a growing number of studies (31). Some studies confirmed in selected cases that FDG PET/CT may represent a useful tool in managing PCa patients (32). As a result, although conventional FDG imaging may currently have poor sensitivity in PCa diagnosis, in this study we found that FDG delayed imaging improves the sensitivity of PCa primary foci diagnosis.

Unlike other studies, this study failed to confirm the correlation between FDG and Gleason score (33, 34), which may be related to the slightly small sample size. We also analyzed the sensitivity of FDG and PSMA at different Gleason scores and in different PSA risk groups. And due to the lack of precision of the results due to the small amount of data, we put this in the supplemental information (**Supplementary Table 1**). In addition, we did not collect the patients’ MRI findings for comparison with PSMA delayed imaging. This is another limitation of this study.

## 5 Conclusions

PSMA PET/CT had a significant advantage over FDG PET/CT in the search for lymph node metastases as well as bone metastatic lesions in PCa. PSMA PET/CT at 3 h p.i. showed most lesions characteristic for primary PCa, and helps to identify suspicious lymph node metastases, also with a higher uptake and contrast than FDG. However, FDG delayed PET/CT imaging is still important in primary PCa diagnosis, especially for hospitals that do not have ^68^Ga-PSMA-11. The 3 h FDG PET/CT delayed imaging significantly improves the detection rate of PCa primary lesions and may find some lesions missed by PSMA. Further research with larger scale is warranted to corroborate its diagnostic performance, especially in comparison to PSMA PET/CT.

## Data Availability

The original contributions presented in the study are included in the article/**Supplementary Material**. Further inquiries can be directed to the corresponding authors.
