# Supplementary material for: Head-to-head comparison of 68Ga-PSMA-11 and 18F-FDG in delayed PET/CT imaging in prostate cancer diagnosis
Source: Front Oncol. 2025 Apr 11;15:1515653. doi: 10.3389/fonc.2025.1515653 (PMC12021635; doi:10.3389/fonc.2025.1515653)
Supplement: Supplementary file 1 [file Table1.docx]

Supplemental table 1. Sensitivity of FDG and PSMA at different Gleason scores and in different PSA risk groups.

|  | ^18^F-FDG PET/CT | | ^68^Ga-PSMA PET/CT | |
| --- | --- | --- | --- | --- |
|  | 1h (normal imaging) | 3h (delayed imaging) | 1h (normal imaging) | 3h (delayed imaging) |
| Gleason score |  |  |  |  |
| 6 | 33.33% | 80% | 100% | 100% |
| 7 | 66.67% | 77.78% | 100% | 92.31% |
| 8 | 20% | 100% | 100% | 100% |
| 9 | 100% | 100% | 100% | 100% |
| PSA |  |  |  |  |
| ≤ 10 | 25% | 76.19% | 100% | 100% |
| 10 - 20 | 47.83% | 64.71% | 86.96% | 86.96% |
| ≥ 20 | 66.67% | 100% | 92% | 100% |

The comprehensive breakdown of Gleason scores and their distribution across patient demographics***.***

| Number | Age | Gleason Score |
| --- | --- | --- |
| 1 | 85 |  |
| 2 | 59 |  |
| 3 | 67 | 2+3 |
| 4 | 67 | 4+3 |
| 5 | 80 | 4+3 |
| 6 | 76 | 4+3 |
| 7 | 77 |  |
| 8 | 51 | 4+4 |
| 9 | 66 |  |
| 10 | 81 | 4+3 |
| 11 | 71 | 3+5 |
| 12 | 72 | 3+3 |
| 13 | 59 |  |
| 14 | 66 | 3+3 |
| 15 | 64 |  |
| 16 | 61 |  |
| 17 | 58 | 4+3 |
| 18 | 74 |  |
| 19 | 68 | 2+3 |
| 20 | 72 | 4+5 |
| 21 | 71 |  |
| 22 | 75 |  |
| 23 | 88 |  |
| 24 | 73 | 2+3 |
| 25 | 53 |  |
| 26 | 79 |  |
| 27 | 67 | 3+3 |
| 28 | 68 |  |
| 29 | 90 |  |
| 30 | 72 | 3+4 |
| 31 | 79 | 3+4 |
| 32 | 70 | 2+2 |
| 33 | 71 |  |
| 34 | 72 | 4+3 |
| 35 | 63 | 3+4 |
| 36 | 66 | 3+4 |
| 37 | 71 |  |
| 38 | 79 |  |
| 39 | 75 |  |
| 40 | 73 |  |
| 41 | 84 |  |
| 42 | 71 |  |
| 43 | 77 |  |
| 44 | 70 | 4+4 |
| 45 | 60 |  |
| 46 | 67 |  |
| 47 | 57 | 3+3 |
| 48 | 68 | 3+3 |
| 49 | 63 | 3+3 |
| 50 | 75 | 4+5 |
| 51 | 75 | 4+4 |
| 52 | 73 |  |
| 53 | 70 |  |
| 54 | 75 | 3+2 |
| 55 | 65 |  |
| 56 | 67 | 3+4 |
| 57 | 67 | 3+4 |
| 58 | 57 |  |
| 59 | 76 |  |
| 60 | 46 |  |
| 61 | 67 |  |
| 62 | 67 |  |
| 63 | 70 |  |
| 64 | 62 |  |
| 65 | 86 |  |
